# Simultaneous optical recording of action potentials and calcium transients in cardiac single cells differentiated from type 1 CPVT-iPS cells

**DOI:** 10.3389/fphys.2025.1579815

**Published:** 2025-06-04

**Authors:** Tadashi Takaki, Norihisa Tamura, Kenichi Imahashi, Tomoyuki Nishimoto, Yoshinori Yoshida

**Affiliations:** ^1^ Department of Cell Growth and Differentiation, Center for iPS Cell Research and Application, Kyoto University, Kyoto, Japan; ^2^ Cardiomyopathy Project, Takeda-CiRA Joint Program (T-CiRA), Fujisawa, Kanagawa, Japan; ^3^ Department of Pancreatic Islet Cell Transplantation, National Institute of Global Health and Medicine, Japan Institute for Health Security, Tokyo, Japan; ^4^ T-CiRA Discovery, Takeda Pharmaceutical Company Limited, Fujisawa, Kanagawa, Japan

**Keywords:** FluoVolt, membrane potential dye, Calbryte 590, calcium transient, catecholaminergic polymorphic ventricular tachycardia, induced pluripotent stem cells, JTV519, KN-93

## Abstract

Numerous reports investigating channelopathies, including Catecholaminergic Polymorphic Ventricular Tachycardia (CPVT), have successfully reproduced using cardiomyocytes (CMs) differentiated from human induced pluripotent stem cells (hiPSCs). However, the relationship between action potentials (AP) and calcium transient waveforms—especially after drug treatment—remains unclear. In this study, we simultaneously loaded a membrane potential dye FluoVolt and the new calcium indicator Calbryte^TM^ 590 AM and optimized stimulation and detection of both dyes to successfully obtain a higher signal-to-noise (S/N) ratio than the conventional membrane potential dye-red fluorescence Ca^2+^ dye combination, thus enabling the simultaneous recording of both AP and calcium transient waveforms in single hiPSC-CMs, which continued even after gradual increases in drug concentration. In drug-loading experiments on CPVT1 (RyR2-I4587V) hiPSC-derived ventricular-like CMs, carvedilol and flecainide demonstrated some effectiveness, while JTV519 at 3 µM exhibited both efficacy and alterations in AP waveforms. The Ca^2+^/calmodulin-dependent serine-threonine protein kinase II (CaMKII) inhibitor KN-93 at 1 µM was highly effective (93%) at reducing Ca^2+^ transient abnormalities without altering AP waveforms.

## Introduction

Human induced pluripotent stem cell-derived cardiomyocytes (hiPSC-CMs) have been successfully used to recapitulate the catecholaminergic polymorphic ventricular tachycardia (CPVT) (GeneReviews 2024) phenotype and evaluate drug effectiveness. However, the simultaneous recording of action potentials and calcium transient waves in single cells has not been routinely performed (Fatima et al., 2011; Itzhaki et al., 2012; Jung et al., 2012; Di Pasquale et al., 2013; Penttinen et al., 2015a; Penttinen et al., 2015b; Preininger et al., 2016; Sasaki et al., 2016; Maizels et al., 2017; Pölönen et al., 2018; Bueno-Levy et al., 2019; Zhang et al., 2021; Gao et al., 2023). We previously reported that a new membrane potential dye, FluoVolt (FV), enables CM subtype classification and early afterdepolarization (EAD) detection in long QT syndrome hiPSC-CMs following optimization (Takaki et al., 2019). Since CMs differentiated from CPVT-iPSCs likely include subtypes other than the ventricular subtype, we hypothesized that simultaneous imaging of membrane potentials and calcium transients would be necessary to analyze CPVT-iPSC-CMs. Before the development of the low phototoxicity membrane potential dye FV, RH237 or Di-8-ANEPPS was used in combination with the red Ca^2+^ dye Rhod-2 or Fura-4F, but it was difficult to capture single-cell images (Lang et al., 2011; Lee et al., 2012). To simultaneously measure membrane potential and Ca^2+^ transients in single cells, transfection using a viral vector such as ArcLight/R-GECO1 was necessary (Song et al., 2015). Although there have been reports of single-cell analysis using a combination of FV and Rhod-2 (Martinez-Hernandez et al., 2022; Yang et al., 2024), the conventional red calcium indicator exhibits higher mitochondrial affinity than cytosolic calcium ions, has a lower signal-to-noise (S/N) ratio, and requires the presence of an organic anion transporter inhibitor (Zhao et al., 2019; Yang et al., 2024). Therefore, we used the recently developed Ca^2+^ fluorescent indicator, Calbryte^TM^ 590 AM (CB590), which is 10 times more sensitive than Rhod-2, in combination with FV and demonstrated the possibility of capturing single-cell images for long periods by reducing phototoxicity while optimizing the LED light intensity for each fluorophore and to observe the response of CPVT-iPSC-CMs to increasing drug concentrations.

## Methods

### Human iPS cells from type1 CPVT patient

This study was approved by the Ethics Committees of the Graduate School of Medicine Kyoto University and the Kyoto University Hospital. Written informed consent was obtained from the patient in accordance with the Declaration of Helsinki. The CPVT1 (RyR2-I4587V) hiPSC line was kindly gifted from Dr. Sasaki (Sasaki et al., 2016). The hiPSC line derived from a healthy donor, 201B7 (Takahashi et al., 2007), was used as a control hiPSC.

### Human iPS cell culture

Human iPSCs were maintained on STO feeder layers cultured with primate ES cell medium (ReproCell, Yokohama, Kanagawa, Japan) as previously described (Takahashi et al., 2007). Please refer to Supplementary Methods for details.

### Cardiac differentiation and fluorescence-activated cell sorting

Cardiac differentiation from hiPSCs was performed by embryoid body (EB) formation in 96-well plates, with reaggregation on day 3. EBs were subsequently transferred to 10 cm dishes on day 7, and FACS sorting was performed on day 30 using the surface marker SIRPA as in previous reports (Miki et al., 2015; Funakoshi et al., 2016; Takaki et al., 2019). Please refer to Supplementary Methods for details.

### Thawing frozen hiPSC-CMs and cell seeding

hiPSC-CMs were thawing from frozen stocks and seeded as previously described (Takaki et al., 2019). Please refer to Supplementary Methods for details.

### Loading of FV and CB590

When loading FluoVolt (Thermo Fisher Scientific) and Calbryte^TM^ 590 AM (AAT Bioquest, Pleasanton, CA, United States), the medium was exchanged with Gey’s Balanced Salt Solution (GBSS, Sigma-Aldrich) containing FV (0.1% volume) and 10 µM CB590. The medium was replaced with GBSS at 40 min after loading.

### Preincubation

One hour before imaging, the 35 mm glass bottom dish on which hiPSC-CMs were seeded was fixed in Stage Top Incubator® (Tokai Hit, Fujinomiya, Japan) and preincubated at 37°C with 5% CO_2_ supplied.

### Setting up the imaging system

To simultaneously excite two wavelengths (490 nm for FV and 580 nm for CB590), the X-Cite Turbo Multi-wavelength LED Illumination System (Excelitas Technologies, Waltham, MA, United States) was used to optimize the fluorescence emission separately. In detail, 2 out of 6 wavelength LED lamps were used: LEDs 3 (460–495 nm, center 475 nm) and 5 (525–610 nm, center 575 nm) were set at 5% and 25%–100% light intensity, respectively, with ND filters to reduce the light by 16 times. The BrightLine® full-multiband filter set FITC/TxRed-A-NTE (Semrock, Rochester, NY, United States), consisting of FF01-479/585-25 (Dual Band Exciter), FF01-524/628-25 (Dual Band Exciter), and FF505/606-Di01-25x36 (Dual Band Dichroic), was used inside the ECLIPSE Ti-E inverted fluorescence microscope (Nikon). The objective lens used was Plan Apo Lambda 10x (Nikon) with a 0.45 numerical aperture. To simultaneously capture fluorescence emission at both wavelengths, an image splitting optics, W-VIEW GEMINI (Hamamatsu Photonics, Hamamatsu, Japan), was used. Filter settings inside were beamsplitter FF580-FDi01-25x36 (Semrock), 536/40 nm BrightLine® single-band bandpass filter FF01-536/40-25 (Semrock), and 631/36 nm BrightLine® single-band bandpass filter FF01-631/36-25 (Semrock).

### Simultaneous dual optical recording of APs and calcium transients from single cells

Images were obtained using an electron multiplying CCD (EMCCD) camera, ImagEM X2 (Hamamatsu Photonics) at 37°C with 5% CO_2_ supplied. Subarray images at a resolution of 512 × 256 pixels were recorded every 16 ms using the AquaCosmos software (Hamamatsu Photonics) for 1 min each. The region of interest (ROI) was defined by manually enclosing the flashing area. Non-beating cells were excluded from the analysis. Data was plotted into graphs using Excel 2019 (Microsoft, Redmond, WA, United States).

### Addition of reagents or drugs

The stock solution was diluted, and 200 µL of the newly diluted solution was added to the top of the 35 mm glass-bottom dish to obtain the final concentration as previously described (Takaki et al., 2019). Please refer to Supplementary Methods for details.

### Type of Ca^2+^ transient abnormalities and responses to drugs

Ca^2+^ transient abnormalities were classified into four types and listed in order of severity: oscillation, triple peaks, double peaks, and plateau abnormality. Cells selected for observing drug responses were ventricular-like cells with normal APD and had abnormal Ca^2+^ transients before drug additions. Normal APD was defined as <1 s as previously reported (Takaki et al., 2019). After drug addition, cells were determined as responders if they had normal Ca^2+^ transients, semi-responders if their Ca^2+^ transients changed to milder phenotypes, and non-responders if their Ca^2+^ transients remained unchanged. Responders and semi-responders were considered drug-responsive cells.

### Statistical analysis

All statistical analyses were verified using Fisher’s exact test by Excel 2019. Values considered statistically significant are denoted as ^∗^ *p* < 0.05 and ^∗∗^ *p* < 0.01. When Fisher’s exact was performed multiple times, the significance of the difference was determined by the Bonferroni method. If more than half the hiPSC-CMs stopped beating, they were excluded from the analysis.

## Results

### Establishing a system to record membrane potentials and calcium transients simultaneously

**FIGURE 1:**
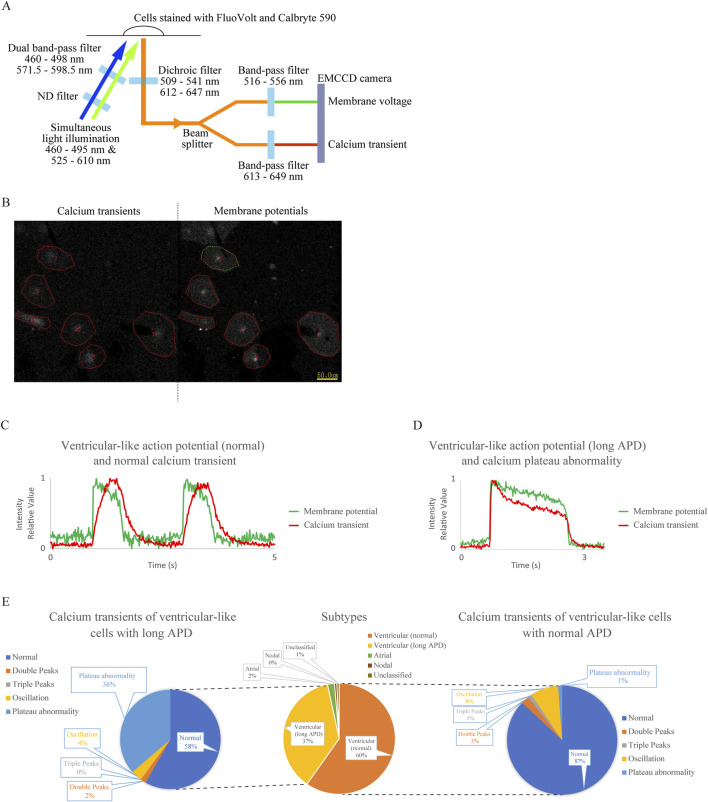
Establishment of a system for simultaneous recording of action potentials (AP) and Ca^2+^ transients using the membrane potential-sensitive dye FluoVolt (FV) and calcium indicator Calbryte 590 (CB590) and analysis of cardiomyocytes differentiated from control human induced pluripotent stem cells (hiPSC-CMs) using this system. **(A)** Schematic diagram of the simultaneous recording system. Two excitation wavelengths of LED light are attenuated to 1/16 intensity by an ND filter and wavelength-limited through a dual bandpass filter before being simultaneously irradiated onto cells stained with the fluorescent dyes. The emitted light at two wavelengths passes through a dichroic filter, is split into two by a beam splitter, and is simultaneously captured by a single electron multiplying CCD (EMCCD) camera through separate bandpass filters. **(B)** Screenshot during recording. Recorded CB590 and FV signals in hiPSC-CMs are on the left and right sides, respectively. Regions of interest (ROIs) were obtained by encircling blinking cells. **(C)** Representative waveform diagram showing membrane potential and Ca^2+^ transient simultaneously recorded from a ventricular-like cell with normal action potential duration (APD). The fluorescent intensity was expressed relative to the minimum value, 0, and the maximum value, 1. **(D)** Representative waveform diagram showing the simultaneous detection of membrane potential and Ca^2+^ transient in a ventricular-like cell with prolonged APD. The APD extended beyond 1 s, and calcium plateau abnormality was observed. The fluorescent intensity was expressed relative to the minimum value, 0, and the maximum value, 1. **(E)** The center panel shows subtype classification based on AP waveforms of control hiPSC-CMs. The right and left panels show classifications of Ca^2+^ transients in ventricular-like cells with normal and long APD, respectively (n = 149). Calcium plateau abnormalities were more frequent in long APD ventricular-like cells than in normal ventricular-like cells (p = 1.87 × 10^*−*8^).

We successfully captured the fluorescence emission from cells stained with the membrane potential dye FV and the calcium indicator CB590 using a single EMCCD camera equipped with separate band-pass filters (Figure 1A). Before setting up this system, we confirmed that the waveforms obtained from hiPSC-CMs stained with FV alone had no leakage into the calcium transient waveforms (Supplementary Figures S1a,b) and that the waveforms obtained from hiPSC-CMs stained with CB590 alone had no leakage into the membrane potential waveforms (Supplementary Figures S1c,d). An example of the image captured during recording is shown in Figure 1B. The sensor is divided into left and right halves. In this example, six hiPSC-CMs flash in response to excitation due to increases in intracellular calcium concentration and changes in membrane potentials on the left and right sides of the screen, respectively. The upper and lower panels of Supplementary Figure S1e show the waveforms of membrane potentials (i.e., AP) and Ca^2+^ transients, respectively. Figure 1C shows an example of normal ventricular APD and Ca^2+^ transient waveforms, and Figure 1D shows an example of a ventricular-like cell with long APD and an abnormal Ca^2+^ transient waveform. Other unusual waveforms from control hiPSC-CMs are shown in Supplementary Figures S1f–l.

Ca^2+^ transient abnormalities are classified into four types based on the literature: double peaks (Supplementary Figure S1h), triple peaks (Supplementary Figure S1i), oscillations (Supplementary Figure S1j), and plateau abnormality (Figure 1D; Supplementary Figure S1g). Their frequencies were analyzed and shown in Figure 1E. Similar to our previous report (Takaki et al., 2019), CM subtypes were classified based on AP waveforms (n = 149), as shown in the center of Figure 1E. Next, Ca^2+^ transients were classified within both normal (right panel in Figure 1E) and long APD ventricular populations (left panel in Figure 1E). Whereas Ca^2+^ transient abnormalities were rare in the former, they were observed more frequently in the latter because calcium plateau abnormalities were more common in the ventricular (long APD) group (p = 1.87 × 10^*−*8^) (Supplementary Table S1).

### Abnormal Ca^2+^ transients in CPVT1 hiPSC-derived ventricular-like CMs

**FIGURE 2:**
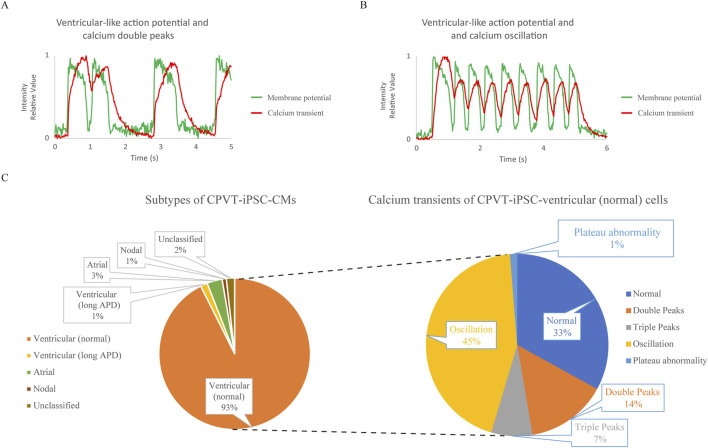
Analysis of cardiomyocytes differentiated from type 1 catecholaminergic polymorphic ventricular tachycardia (CPVT) patient-derived human induced pluripotent stem cells (CPVT1 hiPSC-CMs) using the system to record action potentials (APs) and Ca^2+^ transients simultaneously. **(A)** The waveform of Cell 1 in Supplementary Figure S2a,b. The fluorescence intensity was expressed relative to the minimum value, 0, and the maximum value, 1. Calcium double peaks and ventricular-like AP with normal AP duration (APD) are depicted. **(B)** The waveform of Cell 3 in Supplementary Figure S2a,b. The fluorescent intensity was expressed relative to the minimum value, 0, and the maximum value, 1. Calcium oscillation and ventricular-like AP with normal APD are depicted. **(C)** The left and right pie charts show the subtype classification based on the action potential waveforms of CPVT1 hiPSC-CMs and the classification of Ca^2+^ transients in ventricular-like cells with normal APD, respectively (n = 338). Among the subtypes, most were ventricular-like CMs with normal APD (93%). In their calcium transients, calcium oscillations accounted for a relatively large proportion, with calcium plateau abnormalities only accounting for 1%. Calcium abnormalities accounted for two-thirds of CPVT1 hiPSC-derived ventricular (normal) cells, which was significantly more frequent compared to control hiPSC-derived ventricular (normal) CMs (p = 7.68 × 10^*−*21^).

Next, we also analyzed CPVT1 hiPSC-CMs (Supplementary Figure S2a). The upper and lower panels in Supplementary Figure S2b show AP and Ca^2+^ transient waveforms of the four cells, respectively. The membrane potential and Ca^2+^ transient waveforms of Cell 1 and Cell 3 in Supplementary Figure S2b are shown in Figures 2A,B, respectively. The former represents Ca^2+^ double peaks, while the latter is Ca^2+^ oscillation, both considered typical Ca^2+^ transient abnormalities. The waveforms of Cell 2, as shown in Supplementary Figure S2c, revealed that it is a nodal-like cell that beats very rapidly, indicating that Cell 2 did not exhibit Ca^2+^ oscillations. It has been reported that delayed afterdepolarization (DAD) is a transient inward current due to the exchange of Na^+^ and Ca^2+^ by the sodium-calcium exchanger (NCX) after Ca^2+^ leakage from the sarcoplasmic reticulum (Bers, 2014). In Supplementary Figure S2d, after the action potential ended, the dashed arrow indicated increased calcium intensity due to suspected calcium leakage, followed by an arrow indicating a small increase in membrane potential suggestive of DAD.

We also performed subtyping of CPVT1 hiPSC-CMs (n = 338) and classified Ca^2+^ transients within the normal ventricular-like cell population (Figure 2C). Cardiomyocyte subtyping showed that most cells were normal ventricular-like cells, with two-thirds exhibiting abnormal Ca^2+^ transients, which was significantly higher than those in control hiPSC-derived ventricular-like (normal) cells (p = 7.68 × 10^*−*21^) (Supplementary Table S2), even though calcium plateau abnormalities were observed in only 1%, consistent with control normal hiPSC-derived ventricular-like cells.

### Response of CPVT1 hiPSC-CMs to existing clinical CPVT drugs

**FIGURE 3:**
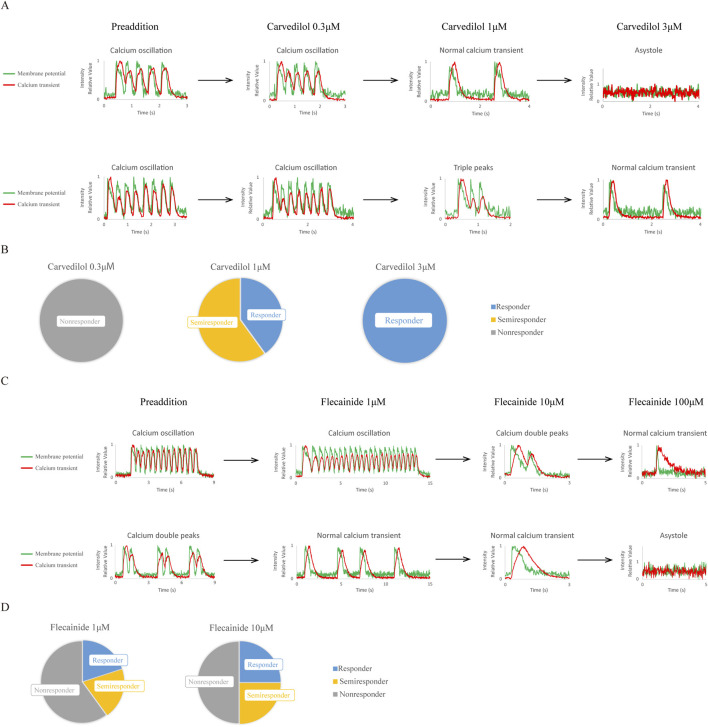
Effects of approved clinical drugs on CPVT1 hiPSC-CMs. **(A)** The upper and lower panels show concentration-dependent changes in action potentials and Ca^2+^ transients induced by nonselective β-blocker carvedilol in two CPVT1 hiPSC-derived ventricular-like cells exhibiting calcium oscillation. Carvedilol at 0.3 µM did not improve calcium abnormalities. At 1 μM, while calcium abnormalities tended to improve (p = 0.0152), some cells showed a shortening of the plateau phase. At 3 μM, while calcium abnormalities were improved (p = 0.0476), some cells stopped beating. **(B)** Analysis of the rate of improvement of Ca^2+^ transient abnormality by carvedilol (n = 5). **(C)** Concentration-dependent changes in action potentials and Ca^2+^ transients induced by a class 1C antiarrhythmic drug, flecainide, in two CPVT1 hiPSC-derived ventricular-like cells, exhibiting calcium oscillation in the upper panel and calcium double peaks in the lower panel. Some cells showed improvement at 1 μM, while another showed a semi-response at 10 µM. **(D)** Analysis of the rate of improvement of Ca^2+^ transient abnormality by flecainide at 1 µM (n = 5) (p = 0.545) and 10 µM (n = 4) (p = 0.545). Cells that stopped beating were excluded from the analysis. Because more than half the cells stopped beating at 100 μM, it was excluded from the analysis.

Next, we tested the response of CPVT hiPSC-CMs to approved clinical drugs, carvedilol (Figures 3A,B; Supplementary Tables S3, S4) and flecainide (Figures 3C,D; Supplementary Tables S5, S6), by increasing concentrations and observing simultaneous changes in membrane potential and Ca^2+^ transients.

Figures 3A,C show the simultaneous recordings of changes in membrane potential and Ca^2+^ transients in two representative cells, with the resulting analysis for all observed cells in Supplementary Tables S3, S5. Figures 3B,D show the responsiveness of cells with calcium abnormalities, and Supplementary Tables S4, S6 show the percentage of cells with normal calcium transient and improved Ca^2+^ abnormalities. The results indicated that carvedilol at 1 and 3 µM improved Ca^2+^ abnormalities (p = 0.0152 and p = 0.0476, respectively), while flecainide at 1 and 10 µM tended to improve them without significant differences. It should be noted that atrial-like and non-beating cells appeared at 3 µM carvedilol and 10 µM flecainide, whereas 100 µM flecainide caused most cells to stop beating and was therefore excluded from the analysis.

### Response of CPVT1 hiPSC-CMs to RyR2 modulator

**FIGURE 4:**
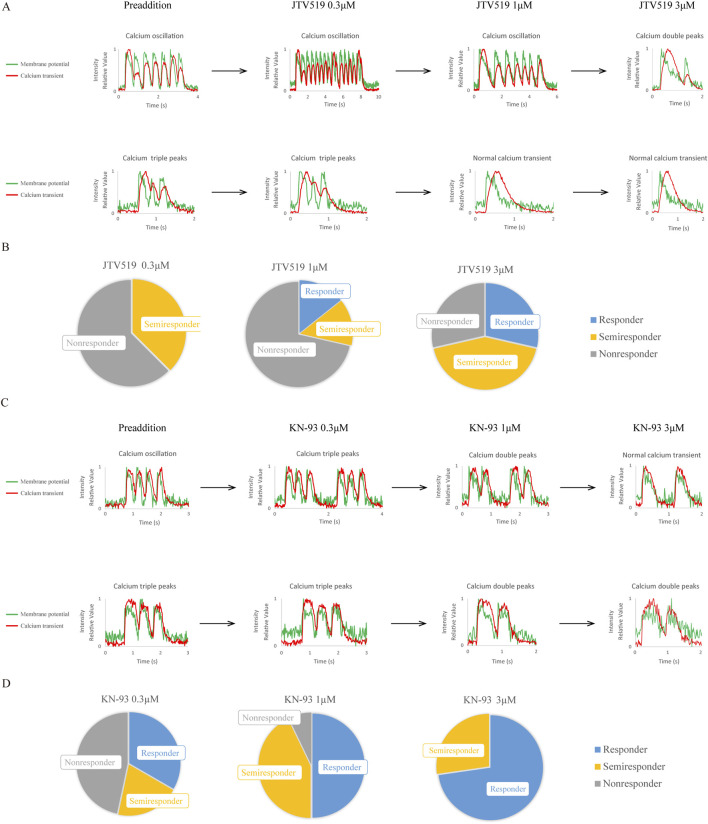
Effects of a RyR2 modulator, JTV519, and a Ca^2+^/calmodulin-dependent serine-threonine protein kinase II (CaMKII) inhibitor, KN-93, on CPVT1 hiPSC-CMs. **(A)** JTV519 (K201)-induced changes in action potentials and Ca^2+^ transients in two CPVT1 hiPSC-derived ventricular-like cells. The upper and lower panels show cells with calcium oscillation and triple peaks before drug additions, respectively. Although Ca^2+^ transient abnormalities tended to improve as the concentration increased, some cells showed a change in action potential to atrial-like at 3 µM. **(B)** Analysis of the rate of improvement of Ca^2+^ transient abnormality by JTV519 (K201) (n = 8). No significant difference at 0.3 (p = 0.350) or 1 µM (p = 0.350). Although a significant difference was observed at 3 µM (p = 0.0230), 80% of the cells displayed atrial-like action potential waveforms. **(C)** KN-93-induced changes in action potentials and Ca^2+^ transients in two CPVT1 hiPSC-derived ventricular-like cells. The upper and lower panels show cells with calcium oscillation and triple peaks before drug additions, respectively. Ca^2+^ transient abnormalities tended to improve as the concentration increased, but some cells showed asystole or changes to atrial-like action potential waveforms at 3 µM. **(D)** Analysis of the rate of improvement of Ca^2+^ transient abnormality by KN-93 at 1 (n = 14) and 3 µM (n = 11). Cells that stopped beating were excluded from the analysis. Ca^2+^ transient abnormalities improved at 0.3, 1, and 3 µM (p = 0.0201, 0.0000660, and 0.000280, respectively).

JTV519 ((4-[3-(4-benzylpiperidin-1-yl)propionyl]-7-methoxy-2,3,4,5-tetrahydro-1,4-benzothiazepine monohydrochloride, K201) is a 1,4-benzothiazepine derivative developed as a cardioprotective drug, previously reported to reduce Ca^2+^ efflux from RyR and improve contractility in failing myocardium (Kohno et al., 2003; Toischer et al., 2010). We thus examined the response of CPVT1 hiPSC-CMs to JTV519 (Figures 4A,B; Supplementary Tables S7, S8). Ca^2+^ transient abnormalities improved in 20% of the cells at 1 μM and 60% at 3 µM. Although there was no significant difference at 0.3 µM (p = 0.350) and 1 µM (p = 0.350), a statistically significant difference was observed at 3 µM (p = 0.0230) (Supplementary Table S8). However, 80% of the cells at 3 µM also displayed atrial-like action potential waveforms (Supplementary Table S7).

### Response of CPVT1 hiPSC-CMs to CaMKII inhibitor

We observed the response of CPVT1 hiPSC-CMs to a CaMKII inhibitor, KN-93 (N-[2-[N-(4-chlorocinnamyl)-N-methylaminomethyl]phenyl]-N-(2-hydroxyethyl)-4-methoxybenzenesulfonamide) and found Ca^2+^ transient abnormalities to improve in a concentration-dependent manner at 0.3, 1, and 3 µM (Figures 4C,D; Supplementary Tables S9, S10) (p = 0.0201, 0.0000660, and 0.000280, respectively). KN-93 at 0.3 µM tended to be less effective on calcium double or triple peaks, but there was no significant difference between calcium oscillation and double or triple peaks (p = 0.277, Supplementary Table S11). Notably, KN-93 at 1 µM was effective in 93% (13/14) of cells without affecting action potentials.

## Discussion

Many studies have demonstrated that single-cell calcium imaging of CPVT-hiPSC-CMs is highly effective in reproducing the pathological condition (Itzhaki et al., 2012; Jung et al., 2012; Penttinen et al., 2015a; Preininger et al., 2016; Bueno-Levy et al., 2019; Zhang et al., 2021; Gao et al., 2023). However, damage during single-cell dispersion, phototoxicity during recording, EAD associated with prolonged APD, and tachycardia caused by nodal or atrial subtypes could be misinterpreted as the CPVT phenotype. To address these issues, we stained hiPSC-CMs with the membrane potential dye FV and the calcium indicator CB590 and optimized two wavelengths of LED light to stimulate each simultaneously. This approach allowed us to classify subtypes and calcium transient abnormalities and observe the dose-dependent effects of several drugs on CPVT-iPSC-CMs.

The first-choice drug for CPVT is a nonselective β-blockers (e.g., propranolol and nadolol), not β1-selective β-blockers (Napolitano et al., 2022; Mazzanti et al., 2022; Peltenburg et al., 2022). Because the patient from which CPVT1 hiPSCs used in this study originated was initially treated with a β1-selective β blocker and the arrhythmia attacks were not controlled, the drug was switched to carvedilol, a nonselective β blocker, which showed some efficacy, however, she continued to experience episodes of ventricular tachycardia, which was prevented by flecainide treatment (Dochi et al., 2007; Sasaki et al., 2016).

Carvedilol is the only β-blocker known to inhibit SR Ca^2+^ store overload induced Ca^2+^ release (SOICR) in HEK293 cells expressing *RyR2*-R4496C regardless of its α-blocking and antioxidant properties and has been shown to reduce RyR2 mean open time and probability (Zhou et al., 2011). In our system, carvedilol at 1 µM induced response or semi-response to Ca^2+^ abnormalities, as in previous studies (Zhou et al., 2011; Maizels et al., 2017; Pölönen et al., 2018), but some cells showed asystole and atrial-like action potentials at 3 µM (Figures 3A,B; Supplementary Tables S3, S4). According to previous literature, the blood concentration of carvedilol is approximately 0.3 µM even when taken orally at a high dose of 25 mg per day (Yilmaz and Arslan, 2016), while the blood concentration has been reported to be approximately 0.06 µM in a patient in Japan (Kitakaze et al., 2012). The blood concentration of carvedilol in this patient did not reach 1 μM, and *in vitro* experiments also suggested that this drug alone could not suppress arrhythmia. However, carvedilol also inhibits phosphorylation of key Ca^2+^ handling proteins by blocking β-stimulatory signaling, thereby potentially preventing arrhythmias (Pölönen et al., 2018), suggesting that continuous oral administration of this drug is of great value. Flecainide binds to the open state of the cardiac sodium channel I_Na_ (Ramos and O’Leary, 2004) but is also known to inhibit I_CaL_, I_to_, I_Kr_, and I_RyR_ (Yang et al., 2021). The proposed therapeutic mechanism of flecainide for CPVT1 involves a direct effect on RyR2 (Kryshtal et al., 2021), an increase in the threshold for triggered activity (TA) (Liu et al., 2011a), and a decrease in I_Na,_ followed by activation of the Na^+^/Ca^2+^ exchanger, thereby reducing the probability of RyR2 opening (Bannister et al., 2015). In our experimental system, flecainide at 1 µM was effective in approximately half the cells, consistent with a previous report on CPVT1 hiPSC-CMs (Pölönen et al., 2018; Zhang et al., 2021). According to the literature, the effective blood concentration of flecainide is about 1.5 µM (Anderson et al., 1981). However, increasing the concentration to 10 µM did not enhance efficacy significantly, suggesting the need for new drug candidates (Figures 3C,D; Supplementary Tables S5, S6). Given that it was effective in three-quarters of CPVT patients (van der Werf et al., 2011), its ability to suppress arrhythmias in about half of the cells *in vitro* may still reflect therapeutic benefit *in vivo*, likely due in part to Na^+^ channel blockade increasing the TA threshold (Liu et al., 2011a; Bannister et al., 2015).

JTV519 (K201) is a 1,4-benzothiazepine derivative that was developed as a cardioprotective drug and was reported to have cardioprotective effects through its inhibitory effect on contractions in a myofibrillar overcontraction model of isolated rat hearts (Kaneko, 1994). It was subsequently shown to block fast Na^+^ current (I_Na_), I_CaL_, and I_K1_ in guinea pig ventricular myocardium (Kimura et al., 1999), as well as inhibit I_Kr_ (Kiriyama et al., 2000), and was reported to reduce Ca^2+^ efflux from RyR and improve contractility in failing myocardium (Kohno et al., 2003; Toischer et al., 2010). By contrast, no inhibitory effect on ventricular arrhythmias was observed in *Ryr2* ^R4496C+/−^ mice (Liu et al., 2006). Recently, a report showed that JTV519 at 2 µM reduced Ca^2+^ sparks in CPVT1 hiPSC-CMs, although changes in APs were not investigated (Zhang et al., 2021). In our system, Ca^2+^ abnormalities were improved in 60% of the cells at 3 µM (Figures 4A,B; Supplementary Tables S7, S8), but 80% of the cells exhibited a transition from ventricular-like to atrial-like AP waveform at this concentration possibly due to I_CaL_ and SERCA inhibition by JTV519 (Loughrey et al., 2007; Sacherer et al., 2012; Darcy et al., 2016). Notably, agents such as carvedilol, flecainide, and JTV519 induced asystole or a change to atrial-like AP at higher concentration, underscoring the clinical need for RyR2 inhibitors that are effective at lower, safer doses (Takenaka et al., 2023; Kurebayashi et al., 2024). In addition, dual-action drugs have been developed that inhibit Ca^2+^ leak from cardiac ryanodine receptors while activating Ca^2+^ uptake via SERCA2 (Wegener et al., 2024).

KN-93, a CaMKII inhibitor, was shown to improve Ca^2+^ abnormalities in the *Ryr2* ^+/R4496C^ KI mouse model of CPVT1 and CPVT1 (RyR2-E2311D)-iPSC-CMs (Liu et al., 2011b; Di Pasquale et al., 2013). We also confirmed that KN-93 was effective even in CPVT-iPSC-CMs with *RYR2* mutation in the transmembrane domain (Figures 4C,D; Supplementary Tables S9, S10). The reason why KN-93, even at low concentrations, had a superior antiarrhythmic effect compared to the three drugs mentioned above is thought to be that it reduces the opening probability by inhibiting RyR2 receptor phosphorylation and CaMKII autophosphorylation, thereby suppressing positive feedback and exerting a sustained effect (Wehrens et al., 2004; Swaminathan et al., 2012). KN-93 at 0.3 µM tended to be less effective on calcium double or triple peaks than oscillation (Supplementary Table S11), likely because it does not directly inhibit RyR2 receptors and is thus incapable of rescuing all calcium abnormalities. Although KN-93 at 1 µM improved all types of calcium abnormalities, CaMKII inhibitors have the disadvantage of acting on other organs (Sałaciak et al., 2021; Chacar et al., 2024; Sun et al., 2024). Therefore, to avoid inhibition of extracardiac CAMKII, attempts have been made to develop peptide or nucleotide drugs and drugs targeting enzymes that act on CaMKII (Reyes Gaido et al., 2023).

This study has some limitations. First, we analyzed hiPSC-CMs with only one *RYR2* mutation. Second, cells were relatively immature. Third, the analysis was performed on single cells without pacing. Thus, there may be variability of condition across cells. Finally, suppressive effects on arrhythmia and side effects at the tissue level were not considered.

## Data Availability

The original contributions presented in the study are included in the article/Supplementary Material, further inquiries can be directed to the corresponding author.
