# Supplementary material for: Simultaneous optical recording of action potentials and calcium transients in cardiac single cells differentiated from type 1 CPVT-iPS cells
Source: Front Physiol. 2025 Jun 4;16:1579815. doi: 10.3389/fphys.2025.1579815 (PMC12175672; doi:10.3389/fphys.2025.1579815)
Supplement: Supplementary file 1 [file Supplementaryfile1.docx]

**Supplementary Methods**

**Human iPS cell culture**

Human iPSCs (hiPSCs) were maintained on STO feeder SL10 cell (ReproCell) layers cultured with primate ES cell medium (ReproCell, Yokohama, Kanagawa, Japan) supplemented with 4 ng/mL recombinant human basic fibroblast growth factor (bFGF, ReproCell) as previously described (Takahashi et al., 2007). For passaging every 7 days, hiPSCs were washed once with PBS and then incubated with Dissociation Solution for human ES/iPS Cells (ReproCell) for 10 min at 37°C. After removing the Dissociation Solution and washing with PBS, primate ES cell medium was added, and cells were detached and collected using a scraper. After centrifugation at 250 × *g* for 5 min, cells were seeded on STO feeder cells at a ratio between 1:4 and 1:5. Feeder cells were seeded according to the manufacturer's protocol.

**Cardiac differentiation and fluorescence-activated cell sorting**

hiPSCs were differentiated by forming embryoid bodies (EBs) as previously described (Miki et al., 2015; Funakoshi et al., 2016; Takaki et al., 2019). On day 29, EBs were exposed to 2mg/mL collagenase I (Sigma-Aldrich, St Louis, MO, USA) in PBS containing 20% fetal bovine serum for 6 hours at 37°C, then exposed to Accumax (Innovative Cell Technologies, San Diego, CA, USA) for 30 min at 37°C, dissociated into single cells by pipetting two to three times, and seeded at 2.5 to 3×10^6^ cells onto a 6-cm dish coated with fibronectin from bovine plasma (Sigma-Aldrich). Seeded cells were collected the following day by Accumax for 10 min at 37°C and subjected to fluorescence-activated cell sorting (FACSAria^TM^ Fusion, BD Biosciences, San Jose, CA, USA). To purify CMs, SIRPa-positive and lineage (CD31, CD49a, CD140b, CD90, or TRA-1-60)-negative cells were sorted (Dubois et al., 2011) and cryopreserved with STEM-CELLBANKER (Nippon Zenyaku Kogyo, Kooriyama, Japan) at -80°C. Cryotubes were transferred to a liquid nitrogen storage tank a few days later.

**Thawing frozen hiPSC-CMs and cell seeding**

The seeding method was similar to a previous report (Takaki et al., 2019). The cryotube was warmed to 37°C using an aluminum beads bath and centrifuged at 300 × *g* for 5 min. Thawed cells were seeded at a density of 10,000 to 20,000 cells/150 µL on the center of the fibronectin-coated 35-mm glass bottom dish (Matsunami, Osaka, Japan) filled with StemPro 34 SFM (Thermo Fisher Scientific, Waltham, MA, USA) supplemented with buffer (2 mM L-glutamine (Thermo Fisher Scientific), 4×10^-4^ M monothioglycerol (MTG) (Sigma-Aldrich), 50 μg/mL ascorbic acid (Sigma-Aldrich), 150 μg/mL transferrin (Roche Diagnostics, Basel, Swiss), 50 U/mL penicillin, 50 μg/mL streptomycin, and 10 ng/mL VEGF (R&D Systems, Minneapolis, MN, USA). Medium (2 mL) was added gently from above the next day to continue culturing and changed every 3–4 days. Cells were subjected to optical recording experiments 6–14 days after seeding.

**Addition of reagents or drugs**

Stock solutions of reagents were prepared in advance and stored at -30°C until use. After recording in 1 mL GBSS without any drug, 200 µL of a drug solution at the desired final concentration was gently added from above, with recording starting 15 min later. To reach the next desired concentration, 200 µL of a drug solution at the next desired final concentration was gently added from above similarly, with recording starting 15 min later. Powder products used were carvedilol (Cayman Chemical, Ann Arbor, MI, USA), flecainide acetate (Sigma-Aldrich), JTV519 hemifumarate (Sigma-Aldrich), and KN-93 (FUJIFILM Wako Pure Chemical). Carvedilol, flecainide, and JTV519 were dissolved in DMSO to prepare stock solutions of 100 mM, 100 mM, and 10 mM, respectively, and KN-93 was dissolved in distilled water to prepare a 10 mM stock solution.

**References**

Dubois, N.C., Craft, A.M., Sharma, P., Elliott, D.A., Stanley, E.G., Elefanty, A.G., et al. (2011). SIRPA is a specific cell-surface marker for isolating cardiomyocytes derived from human pluripotent stem cells. *Nat Biotechnol* 29(11)**,** 1011-1018. doi: 10.1038/nbt.2005.

Funakoshi, S., Miki, K., Takaki, T., Okubo, C., Hatani, T., Chonabayashi, K., et al. (2016). Enhanced engraftment, proliferation, and therapeutic potential in heart using optimized human iPSC-derived cardiomyocytes. *Sci Rep* 6**,** 19111. doi: 10.1038/srep19111.

Miki, K., Endo, K., Takahashi, S., Funakoshi, S., Takei, I., Katayama, S., et al. (2015). Efficient Detection and Purification of Cell Populations Using Synthetic MicroRNA Switches. *Cell Stem Cell* 16(6)**,** 699-711. doi: 10.1016/j.stem.2015.04.005.

Takahashi, K., Tanabe, K., Ohnuki, M., Narita, M., Ichisaka, T., Tomoda, K., et al. (2007). Induction of pluripotent stem cells from adult human fibroblasts by defined factors. *Cell* 131(5)**,** 861-872. doi: 10.1016/j.cell.2007.11.019.

Takaki, T., Inagaki, A., Chonabayashi, K., Inoue, K., Miki, K., Ohno, S., et al. (2019). Optical Recording of Action Potentials in Human Induced Pluripotent Stem Cell-Derived Cardiac Single Cells and Monolayers Generated from Long QT Syndrome Type 1 Patients. *Stem Cells International* 2019**,** 7532657. doi: 10.1155/2019/7532657.
