# Supplementary material for: Simultaneous optical recording of action potentials and calcium transients in cardiac single cells differentiated from type 1 CPVT-iPS cells
Source: Front Physiol. 2025 Jun 4;16:1579815. doi: 10.3389/fphys.2025.1579815 (PMC12175672; doi:10.3389/fphys.2025.1579815)
Supplement: Supplementary file 2 [file Table1.docx]

**Supplementary Tables S1-11.**

**Abbreviations: V (normal), Ventricular with normal APD; APD, action potential duration; V (long APD), Ventricular with long APD; EAD, early afterdepolarization; n.s., No statistical significance was observed.**

**Table S1. Comparison of calcium transient abnormalities between ventricular (normal) and ventricular (long APD) cells differentiated from control hiPSCs**

| **Calcium transient** | **Normal** | **Double peaks** | **Triple peaks** | **Oscillation** | **Plateau abnormality** |
| --- | --- | --- | --- | --- | --- |
| **Ventricular (normal)** | 74 | 2 | 1 | 7 | 1 |
| **Ventricular (long APD)** | 32 | 1 | 0 | 2 | 20 |
| **Significant difference** | - | n.s. | n.s. | n.s. | ** |

Statistical analyses were performed using Fisher's exact test separately between normal calcium transients and double peaks, normal calcium transients and triple peaks, normal calcium transients and oscillation, and normal calcium transients and plateau abnormalities. Using the Bonferroni method, * p<0.0125, ** p<0.0025. Plateau abnormalities were more common in ventricular (long APD) with p=1.87×10^-8^.

**Table S2. Comparison of calcium transient abnormalities between control and CPVT1 hiPSC-derived ventricular (normal) cells**

|  | **Calcium transient** | | **Total cell count** |
| --- | --- | --- | --- |
|  | **Normal** | **Abnormal** |  |
| **Control hiPSC-CMs** | 74 | 11 | 85 |
| **CPVT1 hiPSC-CMs** | 103 | 209 | 312 |
| **Total cell count** | 177 | 220 | 397 |

Statistical analyses were performed using Fisher's exact test for the incidence of normal and abnormal calcium transients between control and CPVT hiPSC-ventricular (normal) cells, with p= 7.68×10^-21^.

**Table S3. Changes in calcium transients and cardiac subtypes by carvedilol treatment**

|  |  | **Carvedilol (µM)** | **0** |  | **0.3** |  | **1** |  | **3** |
| --- | --- | --- | --- | --- | --- | --- | --- | --- | --- |
| **CPVT** | **Calcium transient** | Cell 1 | Oscillation | → | Oscillation | → | Normal | → | Asystole |
|  |  | Cell 2 | Oscillation | → | Oscillation | → | Double peaks | → | Normal |
|  |  | Cell 3 | Oscillation | → | Oscillation | → | Triple peaks | → | Normal |
|  |  | Cell 4 | Oscillation | → | Oscillation | → | Double peaks | → | Asystole |
|  |  | Cell 5 | Oscillation | → | Oscillation | → | Normal | → | Normal |
|  |  | Cell 6 | Normal | → | Normal | → | Asystole | → | Asystole |
|  | **Subtype** | Cell 1 | V (normal) | → | V (normal) | → | V (normal) | → | Asystole |
|  |  | Cell 2 | V (normal) | → | V (normal) | → | V (normal) | → | V (normal) |
|  |  | Cell 3 | V (normal) | → | V (normal) | → | V (normal) | → | V (normal) |
|  |  | Cell 4 | V (normal) | → | V (normal) | → | V (normal) | → | Asystole |
|  |  | Cell 5 | V (normal) | → | V (normal) | → | Atrial | → | Atrial |
|  |  | Cell 6 | V (normal) | → | V (normal) | → | Asystole | → | Asystole |
| **Control** | **Calcium transient** | Cell 1 | Normal | → | Normal | → | Normal | → | Asystole |
|  |  | Cell 2 | Triple Peaks | → | Oscillation | → | Oscillation | → | Normal |
|  |  | Cell 3 | Normal | → | Normal | → | Normal | → | Normal |
|  |  | Cell 4 | Normal | → | Normal | → | Normal | → | Normal |
|  |  | Cell 5 | Plateau abnormality | → | Normal | → | Normal | → | Normal |
|  |  | Cell 6 | Normal | → | Normal | → | Normal | → | Normal |
|  |  | Cell 7 | Normal | → | Double Peaks | → | Normal | → | Normal |
|  | **Subtype** | Cell 1 | V (normal) | → | V (normal) | → | V (normal) | → | Asystole |
|  |  | Cell 2 | V (normal) | → | V (normal) | → | V (normal) | → | V (normal) |
|  |  | Cell 3 | V (long APD) | → | V (normal) | → | V (normal) | → | V (normal) |
|  |  | Cell 4 | V (normal) | → | V (normal) | → | V (normal) | → | V (normal) |
|  |  | Cell 5 | V (long APD) | → | V (long APD) | → | V (long APD) | → | V (normal) |
|  |  | Cell 6 | V (normal) | → | V (normal) | → | V (normal) | → | V (normal) |
|  |  | Cell 7 | V (normal) | → | V (normal) | → | V (normal) | → | V (normal) |

**Table S4. Analysis of calcium transient improvement by carvedilol treatment**

| **Carvedilol concentration (µM)** | **0** | **0.3** | **1** | **3** |
| --- | --- | --- | --- | --- |
| **Percentage of normal calcium transient cells and responsive and semi-responsive cells** | 0 | 16.7% | 100% | 100% |
| **Significant difference** | - | n.s. | * | * |

Statistical analyses were performed using Fisher's exact test between 0 and 0.3 µM, between 0 and 1 µM, and between 0 and 3 µM. *p<0.05. Carvedilol at 1µM and 3 µM significantly improved calcium abnormalities (p=0.0152 and 0.0476, respectively).

**Table S5. Changes in calcium transients and cardiac subtypes by flecainide treatment**

|  |  | **Flecainide (µM)** | **0** |  | **1** |  | **10** |  | **100** |
| --- | --- | --- | --- | --- | --- | --- | --- | --- | --- |
| **CPVT** | **Calcium transient** | Cell 1 | Oscillation | → | Oscillation | → | Double peaks | → | Normal |
|  |  | Cell 2 | Oscillation | → | Oscillation | → | Oscillation | → | Asystole |
|  |  | Cell 3 | Oscillation | → | Oscillation | → | Oscillation | → | Asystole |
|  |  | Cell 4 | Double peaks | → | Normal | → | Normal | → | Asystole |
|  |  | Cell 5 | Oscillation | → | Plateau abnormality | → | No beating | → | Normal |
|  |  | Cell 6 | Normal | → | Normal | → | Plateau abnormality | → | Asystole |
|  | **Subtype** | Cell 1 | V (normal) | → | V (normal) | → | Atrial | → | V (normal) |
|  |  | Cell 2 | V (normal) | → | V (normal) | → | V (normal) | → | Asystole |
|  |  | Cell 3 | V (normal) | → | V (normal) | → | V (normal) | → | Asystole |
|  |  | Cell 4 | V (normal) | → | V (normal) | → | V (normal) | → | Asystole |
|  |  | Cell 5 | V (normal) | → | V (long APD) | → | No beating | → | Unclassified |
|  |  | Cell 6 | V (normal) | → | V (long APD) | → | V (long APD) | → | Asystole |
| **Control** | **Calcium transient** | Cell 1 | Normal | → | Plateau abnormality | → | Triple Peaks | → | Asystole |
|  |  | Cell 2 | Normal | → | Normal | → | Double Peaks | → | Asystole |
|  |  | Cell 3 | Normal | → | Normal | → | Normal | → | Asystole |
|  |  | Cell 4 | Normal | → | Normal | → | Normal | → | Asystole |
|  |  | Cell 5 | Plateau abnormality | → | Normal | → | Normal | → | Asystole |
|  |  | Cell 6 | Normal | → | Normal | → | Normal | → | Asystole |
|  |  | Cell 7 | Plateau abnormality | → | Plateau abnormality | → | Double Peaks | → | Asystole |
|  | **Subtype** | Cell 1 | V (long APD) | → | V (long APD) | → | V (long APD) | → | Asystole |
|  |  | Cell 2 | V (normal) | → | V (normal) | → | V (normal) | → | Asystole |
|  |  | Cell 3 | V (normal) | → | V (normal) | → | Atrial | → | Asystole |
|  |  | Cell 4 | V (normal) | → | V (normal) | → | V (normal) | → | Asystole |
|  |  | Cell 5 | V (long APD) | → | V (long APD) | → | V (normal) | → | Asystole |
|  |  | Cell 6 | V (normal) | → | V (normal) | → | V (normal) | → | Asystole |
|  |  | Cell 7 | V (normal) | → | V (long APD) | → | V (long APD) | → | Asystole |

**Table S6. Analysis of calcium transient improvement by flecainide treatment**

| **Flecainide concentration (µM)** | **0** | **1** | **10** | **100** |
| --- | --- | --- | --- | --- |
| **Percentage of normal calcium transient cells and responsive and semi-responsive cells** | 0 | 50% | 40% | N/A |
| **Significant difference** | - | n.s. | n.s. | N/A |

Statistical analyses were performed using Fisher's exact test between 0 and 1 µM, and between 0 and 10 µM. *p<0.05. 100 µM flecainide was excluded from the statistical analysis because all cells stopped beating.

**Table S7. Changes in calcium transients and cardiac subtypes by JTV519 treatment**

|  |  | **JTV519 (µM)** | **0** |  | **0.3** |  | **1** |  | **3** |
| --- | --- | --- | --- | --- | --- | --- | --- | --- | --- |
| **CPVT** | **Calcium transient** | Cell 1 | Triple Peaks | → | Oscillation | → | Oscillation | → | Double Peaks |
|  |  | Cell 2 | Double Peaks | → | Triple Peaks | → | Oscillation | → | Triple Peaks |
|  |  | Cell 3 | Normal | → | Normal | → | Normal | → | Normal |
|  |  | Cell 4 | Oscillation | → | Oscillation | → | Oscillation | → | Double Peaks |
|  |  | Cell 5 | Oscillation | → | Oscillation | → | Oscillation | → | Double Peaks |
|  |  | Cell 6 | Triple Peaks | → | Plateau abnormality | → | Plateau abnormality | → | Normal |
|  |  | Cell 7 | Oscillation | → | Plateau abnormality | → | Plateau abnormality | → | Plateau abnormality |
|  |  | Cell 8 | Oscillation | → | Plateau abnormality | → | Oscillation | → | Oscillation |
|  |  | Cell 9 | Normal | → | Normal | → | Normal | → | Normal |
|  |  | Cell 10 | Triple Peaks | → | Triple Peaks | → | Normal | → | Normal |
|  | **Subtype** | Cell 1 | V (normal) | → | V (normal) | → | V (normal) | → | Atrial |
|  |  | Cell 2 | V (normal) | → | V (normal) | → | V (normal) | → | Atrial |
|  |  | Cell 3 | V (normal) | → | V (normal) | → | V (normal) | → | Atrial |
|  |  | Cell 4 | V (normal) | → | V (normal) | → | V (normal) | → | Atrial |
|  |  | Cell 5 | V (normal) | → | V (normal) | → | V (normal) | → | Atrial |
|  |  | Cell 6 | V (normal) | → | V (long APD) | → | V (long APD) | → | Atrial |
|  |  | Cell 7 | V (long APD) | → | V (long APD) | → | V (long APD) | → | V (long APD) |
|  |  | Cell 8 | V (normal) | → | V (long APD) | → | V (long APD) | → | V (long APD) |
|  |  | Cell 9 | V (normal) | → | V (normal) | → | V (normal) | → | V (normal) |
|  |  | Cell 10 | V (normal) | → | V (normal) | → | V (normal) | → | V (normal) |
| **Control** | **Calcium transient** | Cell 1 | Normal | → | Triple Peaks | → | Asystole | → | Asystole |
|  |  | Cell 2 | Plateau abnormality | → | Plateau abnormality | → | Plateau abnormality | → | Normal |
|  |  | Cell 3 | Normal | → | Double Peaks | → | Normal | → | Normal |
|  |  | Cell 4 | Normal | → | Normal | → | Normal | → | Normal |
|  |  | Cell 5 | Oscillation | → | Oscillation | → | Oscillation | → | Double Peaks |
|  |  | Cell 6 | Plateau abnormality | → | Plateau abnormality | → | Plateau abnormality | → | Triple Peaks |
|  |  | Cell 7 | Normal | → | Normal | → | Asystole | → | Asystole |
|  | **Subtype** | Cell 1 | V (normal) | → | Atrial | → | Asystole | → | Asystole |
|  |  | Cell 2 | V (long APD) | → | V (long APD) | → | V (long APD) | → | Atrial |
|  |  | Cell 3 | V (normal) | → | V (normal) | → | V (normal) | → | Atrial |
|  |  | Cell 4 | V (long APD) | → | V (normal) | → | V (normal) | → | Atrial |
|  |  | Cell 5 | V (long APD) | → | V (long APD) | → | V (long APD) | → | V (normal) |
|  |  | Cell 6 | V (long APD) | → | V (long APD) | → | V (long APD) | → | V (long APD) |
|  |  | Cell 7 | V (normal) | → | V (normal) | → | Asystole | → | Asystole |

**Table S8. Analysis of calcium transient improvement by JTV519 treatment**

| **JTV519 concentration (µM)** | **0** | **0.3** | **1** | **3** |
| --- | --- | --- | --- | --- |
| **Percentage of normal calcium transient cells and responsive and semi-responsive cells** | 20% | 50% | 50% | 80% |
| **Significant difference** | - | n.s. | n.s. | * |

Statistical analyses were performed using Fisher's exact test between 0 and 0.3 µM, 0 and 1 µM, and 0 and 3 µM. *p<0.05. A cell with long APD at 0 µM was excluded from the analysis. JTV519 at 3 µM significantly improved calcium abnormalities (p=0.0230).

**Table S9. Changes in calcium transients and cardiac subtypes by KN-93 treatment**

|  |  | **KN-93 (µM)** | **0** |  | **0.3** |  | **1** |  | **3** |
| --- | --- | --- | --- | --- | --- | --- | --- | --- | --- |
| **CPVT** | **Calcium transient** | Cell 1 | Oscillation | → | Normal | → | Normal | → | Asystole |
|  |  | Cell 2 | Oscillation | → | Double Peaks | → | Normal | → | Normal |
|  |  | Cell 3 | Oscillation | → | Normal | → | Normal | → | Asystole |
|  |  | Cell 4 | Double Peaks | → | Normal | → | Normal | → | Asystole |
|  |  | Cell 5 | Oscillation | → | Normal | → | Asystole | → | Asystole |
|  |  | Cell 6 | Oscillation | → | Oscillation | → | Double Peaks | → | Normal |
|  |  | Cell 7 | Double Peaks | → | Normal | → | Normal | → | Normal |
|  |  | Cell 8 | Plateau abnormality | → | Plateau abnormality | → | Plateau abnormality | → | Normal |
|  |  | Cell 9 | Oscillation | → | Oscillation | → | Normal | → | Normal |
|  |  | Cell 10 | Normal | → | Normal | → | Plateau abnormality | → | Plateau abnormality |
|  |  | Cell 11 | Normal | → | Normal | → | Normal | → | Normal |
|  |  | Cell 12 | Double Peaks | → | Double Peaks | → | Normal | → | Normal |
|  |  | Cell 13 | Normal | → | No beating | → | Normal | → | Normal |
|  |  | Cell 14 | Oscillation | → | Triple Peaks | → | Double Peaks | → | Normal |
|  |  | Cell 15 | Oscillation | → | Triple Peaks | → | Double Peaks | → | Normal |
|  |  | Cell 16 | Triple Peaks | → | Triple Peaks | → | Double Peaks | → | Double Peaks |
|  |  | Cell 17 | Normal | → | Normal | → | No beating | → | Normal |
|  |  | Cell 18 | Triple Peaks | → | Triple Peaks | → | Double Peaks | → | Double Peaks |
|  |  | Cell 19 | Triple Peaks | → | Triple Peaks | → | Double Peaks | → | Double Peaks |
|  | **Subtype** | Cell 1 | V (normal) | → | V (normal) | → | V (normal) | → | Asystole |
|  |  | Cell 2 | V (normal) | → | V (long APD), EAD | → | V (long APD), EAD | → | Atrial |
|  |  | Cell 3 | V (normal) | → | V (normal) | → | V (normal) | → | Asystole |
|  |  | Cell 4 | V (normal) | → | V (normal) | → | V (normal) | → | Asystole |
|  |  | Cell 5 | V (normal) | → | V (normal) | → | Asystole | → | Asystole |
|  |  | Cell 6 | V (normal) | → | V (normal) | → | V (long APD), EAD | → | Atrial |
|  |  | Cell 7 | V (normal) | → | V (normal) | → | V (normal) | → | V (long APD), EAD |
|  |  | Cell 8 | V (normal) | → | V (normal) | → | V (normal) | → | V (normal) |
|  |  | Cell 9 | V (normal) | → | V (normal) | → | V (normal) | → | V (normal) |
|  |  | Cell 10 | V (normal) | → | V (normal) | → | V (normal) | → | V (long APD), EAD |
|  |  | Cell 11 | V (normal) | → | V (normal) | → | V (normal) | → | V (normal) |
|  |  | Cell 12 | V (normal) | → | V (normal) | → | V (normal) | → | V (normal) |
|  |  | Cell 13 | V (normal) | → | No beating | → | V (normal) | → | V (normal) |
|  |  | Cell 14 | V (normal) | → | V (normal) | → | V (normal) | → | V (normal) |
|  |  | Cell 15 | V (normal) | → | V (normal) | → | V (normal) | → | V (normal) |
|  |  | Cell 16 | V (normal) | → | V (normal) | → | V (normal) | → | V (normal) |
|  |  | Cell 17 | V (normal) | → | V (normal) | → | No beating | → | V (normal) |
|  |  | Cell 18 | V (normal) | → | V (normal) | → | V (normal) | → | V (normal) |
|  |  | Cell 19 | V (normal) | → | V (normal) | → | V (normal) | → | V (normal) |
| **Control** | **Calcium transient** | Cell 1 | Normal | → | Normal | → | Normal | → | Double Peaks |
|  |  | Cell 2 | Normal | → | Normal | → | Normal | → | Normal |
|  |  | Cell 3 | Normal | → | Normal | → | Normal | → | Normal |
|  |  | Cell 4 | Plateau abnormality | → | Plateau abnormality | → | Plateau abnormality | → | Normal |
|  |  | Cell 5 | Plateau abnormality | → | Plateau abnormality | → | Triple Peaks | → | Normal |
|  |  | Cell 6 | Normal | → | Plateau abnormality | → | Plateau abnormality | → | Normal |
|  |  | Cell 7 | Normal | → | Normal | → | Normal | → | Normal |
|  |  | Cell 8 | Normal | → | Plateau abnormality | → | Plateau abnormality | → | Normal |
|  |  | Cell 9 | Plateau abnormality | → | Normal | → | Normal | → | Normal |
|  |  | Cell 10 | Normal | → | Plateau abnormality | → | Normal | → | Normal |
|  |  | Cell 11 | Plateau abnormality | → | Plateau abnormality | → | Normal | → | Normal |
|  |  | Cell 12 | Plateau abnormality | → | Plateau abnormality | → | Double Peaks | → | Normal |
|  |  | Cell 13 | Normal | → | Normal | → | Normal | → | Asystole |
|  |  | Cell 14 | Normal | → | Plateau abnormality | → | Normal | → | Asystole |
|  |  | Cell 15 | Normal | → | Normal | → | Normal | → | Asystole |
|  | **Subtype** | Cell 1 | V (normal) | → | V (normal) | → | V (normal) | → | V (long APD), EAD |
|  |  | Cell 2 | V (normal) | → | V (normal) | → | V (normal) | → | V (long APD), EAD |
|  |  | Cell 3 | V (normal) | → | V (normal) | → | V (normal) | → | V (long APD), EAD |
|  |  | Cell 4 | V (long APD) | → | V (long APD) | → | V (long APD) | → | V (normal) |
|  |  | Cell 5 | V (long APD) | → | V (long APD) | → | V (long APD), EAD | → | V (normal) |
|  |  | Cell 6 | V (normal) | → | V (normal) | → | V (long APD), EAD | → | V (normal) |
|  |  | Cell 7 | V (normal) | → | V (normal) | → | V (normal) | → | V (normal) |
|  |  | Cell 8 | V (normal) | → | V (long APD) | → | V (long APD) | → | V (long APD) |
|  |  | Cell 9 | V (normal) | → | V (normal) | → | V (normal) | → | V (normal) |
|  |  | Cell 10 | V (normal) | → | V (long APD), EAD | → | V (normal) | → | Nodal |
|  |  | Cell 11 | V (normal) | → | V (long APD) | → | V (long APD) | → | V (normal) |
|  |  | Cell 12 | V (long APD) | → | V (long APD), EAD | → | V (long APD), EAD | → | Atrial |
|  |  | Cell 13 | V (long APD) | → | V (long APD) | → | V (normal) | → | Asystole |
|  |  | Cell 14 | V (normal) | → | V (norma), EAD | → | Atrial | → | Asystole |
|  |  | Cell 15 | V (normal) | → | V (normal) | → | V (normal) | → | Asystole |

**Table S10. Analysis of calcium transient improvement by KN-93 treatment**

| **KN-93 (µM)** | **0** | **0.3** | **1** | **3** |
| --- | --- | --- | --- | --- |
| **Percentage of normal calcium transient cells and responsive and semi-responsive cells** | 0 | 61.1% | 88.2% | 92.3% |
| **Significant difference** | - | * | ** | ** |

Statistical analyses were performed using Fisher's exact test between 0 and 0.3 µM, 0 and 1 µM, and 0 and 3 µM. *p<0.05, **p<0.01. KN-93 at 0.3 µM, 1µM and 3 µM significantly improved calcium abnormalities (p=0.0201, 0.0000660 and 0.000280, respectively).

**Table S11. Analysis of the improvement rate of different types of calcium transient abnormalities by KN-93 treatment (0.3 µM)**

| **Calcium abnormality type at 0 µM** | **Effective at 0.3 µM** | **Ineffective at 0.3 µM** |
| --- | --- | --- |
| **Oscillation** | 75% | 25% |
| **Double or triple peaks** | 33% | 67% |

Statistical analyses were performed using Fisher's exact test between Oscillation and double or triple peaks. No significant difference was observed (p=0.277).
