# Supplementary figures and images for: Simultaneous optical recording of action potentials and calcium transients in cardiac single cells differentiated from type 1 CPVT-iPS cells
Source: Front Physiol. 2025 Jun 4;16:1579815. doi: 10.3389/fphys.2025.1579815 (PMC12175672; doi:10.3389/fphys.2025.1579815)

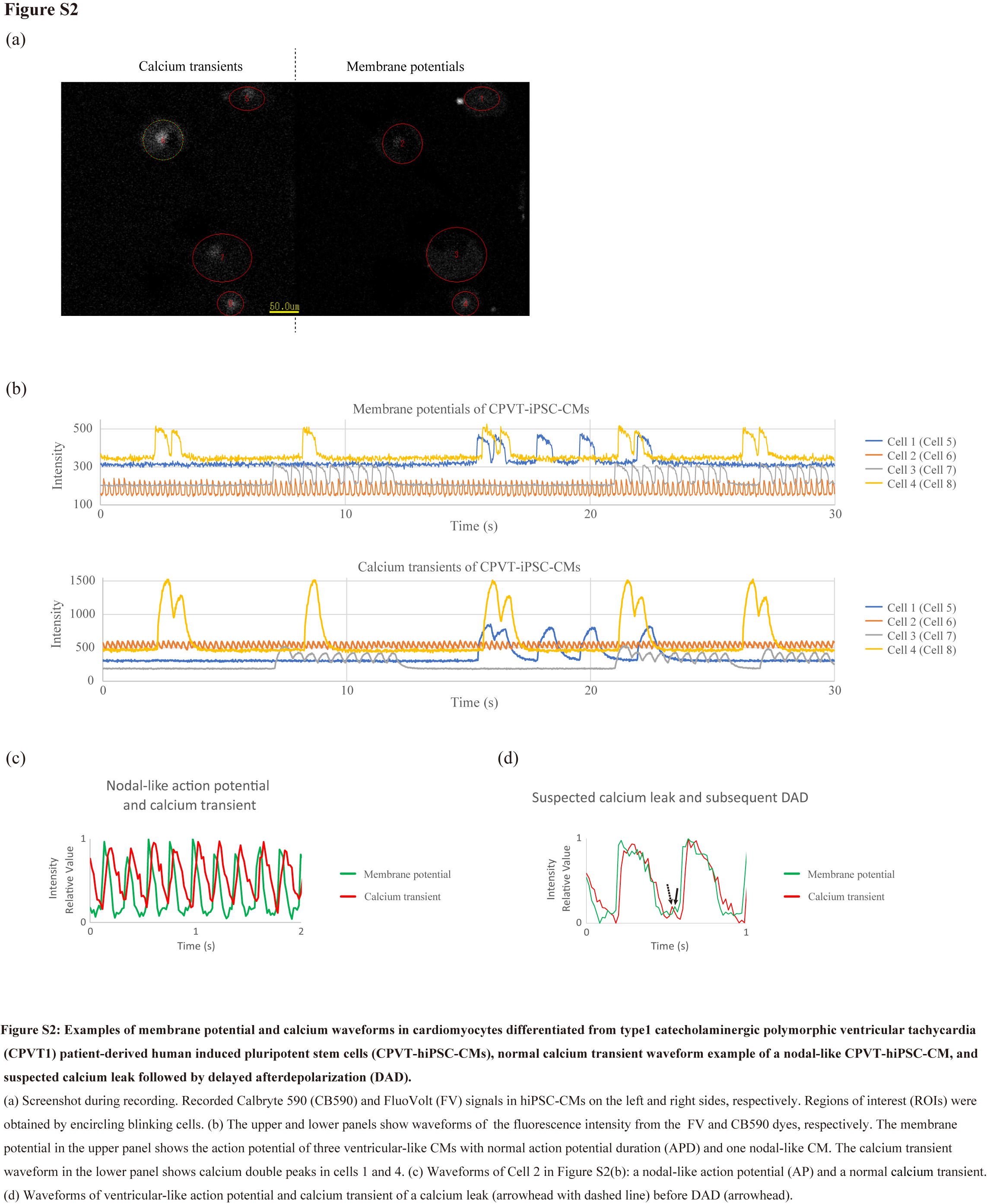

Supplement: Supplementary file 3 [file Image2.tif]

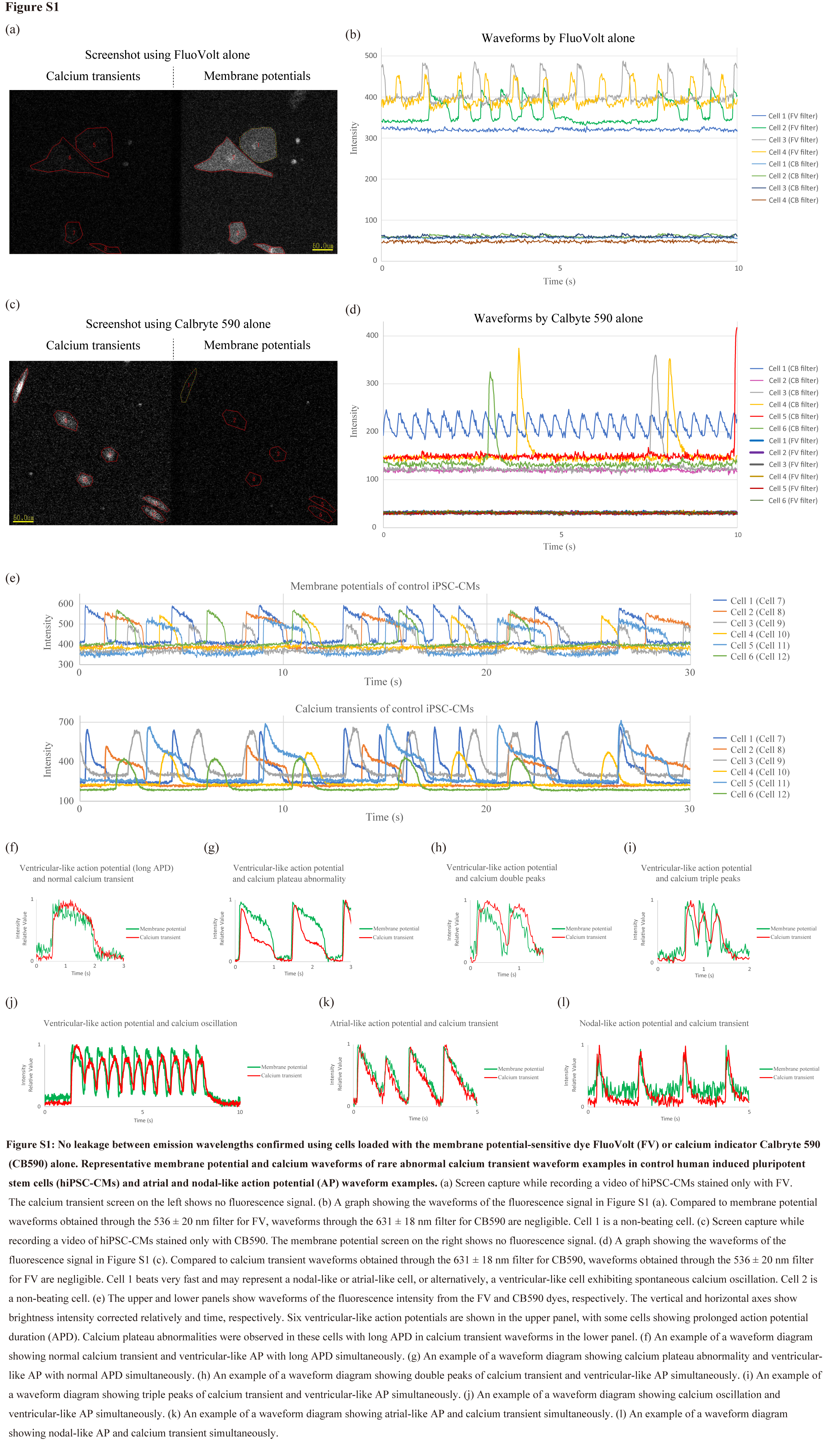

Supplement: Supplementary file 4 [file Image1.tif]
